# Pain phenotypes classified by machine learning using electroencephalography features

**DOI:** 10.1016/j.neuroimage.2020.117256

**Published:** 2020-08-29

**Authors:** Joshua Levitt, Muhammad M. Edhi, Ryan V. Thorpe, Jason W. Leung, Mai Michishita, Suguru Koyama, Satoru Yoshikawa, Keith A. Scarfo, Alexios G. Carayannopoulos, Wendy Gu, Kyle H. Srivastava, Bryan A. Clark, Rosana Esteller, David A. Borton, Stephanie R. Jones, Carl Y. Saab

**Affiliations:** a Department of Neurosurgery, Rhode Island Hospital, Providence, RI, United States; b Department of Neuroscience, Brown University, Providence, RI, United States; c Laboratory for Pharmacology, Asahi Kasei Pharma Corporation, Mifuku, Shizuoka, Japan; d Boston Scientific Neuromodulation, Valencia, CA, United States

## Abstract

Pain is a multidimensional experience mediated by distributed neural networks in the brain. To study this phenomenon, EEGs were collected from 20 subjects with chronic lumbar radiculopathy, 20 age and gender matched healthy subjects, and 17 subjects with chronic lumbar pain scheduled to receive an implanted spinal cord stimulator. Analysis of power spectral density, coherence, and phase-amplitude coupling using conventional statistics showed that there were no significant differences between the radiculopathy and control groups after correcting for multiple comparisons. However, analysis of transient spectral events showed that there were differences between these two groups in terms of the number, power, and frequency-span of events in a low gamma band. Finally, we trained a binary support vector machine to classify radiculopathy versus healthy subjects, as well as a 3-way classifier for subjects in the 3 groups. Both classifiers performed significantly better than chance, indicating that EEG features contain relevant information pertaining to sensory states, and may be used to help distinguish between pain states when other clinical signs are inconclusive.

## 1. Introduction

Pain is a multidimensional sensory, emotional and cognitive experience mediated by distributed neural networks in the brain (Legrain et al., 2011). Mapping these networks and understanding their relation to pain requires a macroscale recording technique operating at a fast sampling rate in complement with a high-dimensional analytical approach (Saab and Barrett, 2016). Electroencephalography (EEG) and advanced statistical tools such as machine learning meet these requirements and offer several advantages over other forms of neuroimaging approaches, including ease of use, efficiency, affordability, and minimal interruption of the standard of care in clinical studies (Levitt and Saab, 2019). In light of the current pain epidemic in the United States and the related opioid crisis, studying the neural correlates of pain is of high clinical relevance, potentially leading to more reliable diagnostic criteria and effective therapies (Davis et al., 2017).

Previous data suggest that resting state EEG power in the low frequency range (3–30 Hz) localized to primary somatosensory neocortex (S1) is enhanced in patients with chronic pain, presumably due to dysrhythmic communication in the thalamocortical network (Schulman et al., 2005; Sarnthein and Jeanmonod, 2008; Walton and Llinás, 2011). In rodent pre-clinical models of chronic pain, theta power (4–8 Hz) in S1 is bidirectionally correlated with pain and analgesia (LeBlanc et al., 2016). Pain-induced theta power in S1 is down-regulated by optogenetic stimulation of GABA neurons in thalamic reticular nucleus, leading to anti-nociceptive behavioral effects (LeBlanc et al., 2017). However, with regard to clinical data, several caveats undermine the interpretation of these results due to the failure to control for variables known to bias EEG analysis including age (Hughes and Cayaffa, 1977; Nguyen et al., 2013; Vlahou et al., 2014; Zoubi et al., 2018), gender (Hu, 2018; Malon et al., 2018; van Putten et al., 2018), observer-dependent identification of EEG artifacts (Levitt et al., 2018), and statistical multiple comparisons (Curran-Everett, 2000), which is often the case for high dimensional EEG data. Moreover, studies investigating the effects of pain on EEG power have focused on mean power in relatively long duration epochs (Sarnthein et al., 2006; Stern et al., 2006; Jensen et al., 2013; Misra et al., 2017). This conventional type of power analysis overlooks the possibility of power fluctuation within a band emerging as transient ‘events’ in the millisecond range. In fact, transient beta events in S1 have been shown to be functionally relevant for sensory detection (Shin et al., 2017).

In this study, we record EEG from human subjects and use first order statistical tests and machine learning methods while carefully controlling for variables listed above. First, we test the hypothesis that mean EEG power in multiple low frequency bands, as well as transient power events within these bands, are modulated in patients with chronic pain compared to healthy subjects. Second, we test the hypothesis that information represented by EEG data is sufficient for training a support vector machine (SVM) algorithm to predict ‘no-pain’ versus ‘high-pain’ states, and to distinguish between different ‘high-pain’ states. Contrary to published literature (Pinheiro et al., 2016), however, our results show that correcting for multiple comparisons, mean power is not statistically different between pain patients and healthy subjects, whereas low-gamma transients events are significantly altered. Moreover, an SVM classifier based on principal components generated from EEG features predicts healthy and pain states with high accuracy.

## 2. Methods

### 2.1. Recruitment of subjects & experimental design

Human subjects were recruited under the supervision of the Rhode Island Hospital institutional review board. 20 subjects were recruited with the following inclusion criteria: 1) A diagnosis of chronic lumbar radiculopathy 2) age 25 years or older 3) in treatment for at least 3 months 4) a VAS (Visual Analog Scale) score greater than or equal to 5 on a 0–10 scale. These subjects were then matched in gender and age with 20 healthy individuals, none of whom reported chronic back pain. None of the subjects in this study had been prescribed opioids.

After obtaining consent, a researcher then fitted the subject to the EEG headset. EEG recordings were performed using the Statnet headset and MicroEEG amplifier system (BioSignal Group, Acton, MA), an FDA-approved portable EEG platform (Ladino et al., 2016). The StatNet has 16 recording channels, as well as a common ground and reference. A sample rate of 250 Hz was used for this portion of the study. Once the EEG system was properly fitted, subjects were asked to sit, facing a blank wall, with their hands on their thighs. Subjects were prompted by the researcher to remain in this position and to alternate between eyes-open and eyes-closed every 60 s for 5 min. In addition to these 40 subjects, 17 more were recruited with a history of chronic pain (pre-SCS group) who were scheduled to receive treatment with an implanted thoracic spinal cord stimulator (SCS) for lumbar pain, with or without radiculopathy, recalcitrant to more conservative measures. Demographic information is summarized in table 1.

### 2.2. EEG preprocessing

All preprocessing, feature extraction, statistics, and machine learning were done using MatLab (MathWorks, Natick, MA). EEGs from the pre-SCS group were collected at a sample rate of 500 Hz, while the EEGs from the healthy and radiculopathy groups were collected at 250 Hz. In order to eliminate data collection bias, a low pass filter with a passband frequency of 120 Hz was applied to all recordings, then the pre-SCS recordings were downsampled to 250 Hz. A high-pass filter with a passband frequency of 1.5 Hz and a notch filter with a stopband of 57–63 Hz were then applied to all recordings. Next, all EEGs were visually inspected using Spike2 (Cambridge Electronic Design, Cambridge, UK) to ascertain if individual channels were of insufficient quality to be included in further analysis. All channels that were not considered to be of high quality were removed, and not included in the following methods and results. 11% of all channels were excluded in this manner. Moreover, every remaining channel was divided into 1-second epochs, and each epoch was tested for the presence of artifacts using a previously validated method based on automated detection of artifacts by a SVM (Levitt et al., 2018). Epochs not containing artifacts which were collected from eyes-open portions of the recordings were included in further analysis; all other epochs were excluded (Supplementary Table 1).

### 2.3. Feature extraction

From the remaining epochs of each recording, the following features were calculated: 1) bandwise PSD (Power Spectral Density) for all channels, 2) bandwise coherence for all channel pairs, and 3) bandwise PAC (Phase-Amplitude Coupling) for all channels.

To create the bandwise PSD for each channel, a periodogram was gathered from every eyes-open, artifact-free epoch, and then these periodograms were averaged together for each channel within each subject. These averaged periodograms were normalized by dividing each frequency bin by the sum of all bins from 3 to 30 Hz. The normalized PSD was used to calculate the bandwise PSD by taking the average of all bins within each of the following four frequency bands: Theta (4–8 Hz), Alpha (9–12 Hz), Beta (13–30 Hz), and Low Gamma (31–50 Hz). This yielded 4 PSD features for every channel included, and a maximum of 64 PSD features per subject (4 bands × 16 channels).

Bandwise coherence was calculated from each of 120 unique channel pairs, by averaging the coherence (MatLab function *mscohere* ) from each epoch that was artifact-free during eyes-open recordings for both of the channels in a given pair. This average coherence was divided into four bands in the same manner as PSDs. This yielded 4 coherence features for each channel pair, and a maximum of 480 coherence features per subject (4 bands × 120 channel pairs, coherence values were not computed from channel pairs for which one or both of the channels included artifacts).

PAC was calculated using the Modulation Index (MI) method (Tort et al., 2010). The center frequencies used for phase included all the even numbers from 2 to 20. The center frequencies used for amplitude included all multiples of 3 from 30 to 54. MI was measured for each pair of phase and amplitude frequencies (90 total pairs) for each channel, including only those timepoints for which there were 5 or more consecutive artifact-free eyes-open epochs. This yielded a 9 × 10 MI matrix, for every channel of every subject, with each row corresponding to one phase center frequency, and each column corresponding to one amplitude center frequency. This MI matrix was converted to bandwise PAC for the following 3 pairs of bands: Theta—Low Gamma, Alpha—Low Gamma, and Beta—Low Gamma. For these purposes, we defined Theta and Alpha as before, Beta as 14–20 Hz, and Gamma as 30–54 Hz. This conversion was accomplished by averaging across the appropriate regions of the MI matrix. This yielded 3 PAC features for each channel, and a maximum of 48 PAC features per subject (3 band-pairs × 16 channels).

### 2.4. Statistical analyses

We used unpaired two-tailed T-tests to compare the bandwise PSDs from the healthy and radiculopathy groups, for each of the 4 bands and 16 channels (64 tests in total). We then used Bonferroni’s correction for multiple comparisons with a family size of 64 to compute corrected P-values for each of these tests. We used two-tailed Wilcoxon rank-sum tests to compare the bandwise coherence from the two groups for each of the 4 bands and 120 channel pairs (480 tests in total), then applying Bonferroni’s correction as above with a family size of 480. Finally, we used two-tailed Wilcoxon rank-sum tests to compare the bandwise PAC from the two groups for each of 3 band pairs and 16 channels (48 tests in total), finally applying Bonferroni’s correction as above with a family size of 48. We chose to use nonparametric statistical tests for both the coherence and PAC because these values are constrained between 0 and 1, and are therefore less likely to follow a normal distribution, as required by Student’s T-test (Seymour et al., 2017). To test for equivalence between PSD, coherence, and PAC features of the healthy and radiculopathy groups, and to set an upper limit on any observed effects, we used the Two One-Sided *t*-test (TOST) procedure, with an equivalence interval of +/− one standard deviation of the healthy distribution for each feature (Rogers et al., 1993; Anisha, 2020)

### 2.5. Spectral event analysis

Building our spectral events analysis approach from the methods of Shin et al. (Shin et al., 2017), spectral events were defined as the maximum power within each closed region of supra-threshold time-frequency space confined to a frequency band-of-interest (BOI). Furthermore, the event features, including event counts/epoch, event power maximum, event duration, and event frequency span (F-span) were defined in the same way as by Shin et al. and were found on an epoch-by-epoch basis. Prior to identifying individual spectral events, however, we sought to empirically define frequency BOIs that might contain differing event probabilities for each feature between the radiculopathy and healthy control groups. BOIs were determined separately for each age-matched pair of radiculopathy/healthy subjects on the complement set of subjects (i.e., on all subjects except for the subjects of the specified pair). This allowed us to determine a BOI for spectral event analysis of each age-matched pair that was independent of the spectral event data contained in a specific pair. For BOI selection, we first calculated the spontaneous time-frequency response (sTFR) at 6–50 Hz by convolving each single-epoch time-series with a Morlet wavelet (width = 5) and normalized each spectrogram by the median PSD for each frequency bin. Note that the median PSD was calculated across the aggregate of time samples (i.e., including all epochs) of a subject for a given channel. Averaging the sTFR for each subject, separately, over epochs and time, we computed the spontaneous PSD (sPSD, i.e., the median-normalized PSD) and compared the radiculopathy to the healthy normal group in a two-tailed Wilcoxon rank-sum test at each frequency bin (with Bonferroni correction for multiple comparisons at a family-wise error rate of 0.05). Frequency values deemed significant and that formed a continuous BOI with greater than two bins were passed into the spectral event-finding algorithm in the *SpectralEvents* toolbox (https://github.com/jonescompneurolab/SpectralEvents, findMethod = 3) as the BOI. Two-tailed Wilcoxon signed-rank test was performed on the pairwise differences between mean spectral event features across age-matched radiculopathy/healthy subject pairs.

Note that while the search for a frequency BOI was conducted for 5 of the 16 EEG channels (C3, CZ, F8, FP1, and FP2), significant positive results for a BOI in more than 50% of the age-matched radiculopathy/healthy subject pairs only occured at C3 and CZ. The five aforementioned channels were selected because they passed visual inspection of the raw time-series for all 40 subjects. Following rejection of all 1-second time bins containing artifacts (identified upon visual inspection and the use of an automated SVM algorithm described above), and all single, isolated, artifact-free epochs, 4 of the 20 radiculopathy subjects, along with their age/gender-matched counterparts, were also discarded from spectral event analysis in channels FP1 and FP2 because they contained fewer than 10 artifact-free epochs.

### 2.6. Classification and prediction

The features described above (bandwise PSDs, bandwise coherence and PAC— note that for purposes of this portion of the analysis, we used the full MI matrices instead of bandwise PAC) from the radiculopathy and healthy groups were used to create a feature-set for training a classification SVM. However, since this dataset had far more features than samples, as well as highly unequal numbers of features within each of the three categories (64 for PSD, 480 for coherence, and 1440 for PAC), and some missing features due to channels being rejected by visual inspection, additional preprocessing was required. First, to appropriately replace each missing feature, hot deck imputation was used (Joenssen and Bankhofer, 2012) whereby missing values are filled in with ‘donor’ values from other samples within the dataset selected by identifying the donor sample that is closest to the original sample in feature space (MatLab function *knnimpute* ). Next, principal component analysis (PCA) was performed to reduce the number of features from each of the 3 categories, and the 11 features which explained the greatest variance were used. This number was chosen using a grid search with optimization metric being the accuracy score of the final classifier. PCA was conducted separately on each of the 3 feature sets (PSDs, coherence, and PAC) so that each feature set yielded exactly 11 PCA features, which were independent of the other 2 feature sets. This ensured both that each feature set had the same contribution to the classification feature set, and that we had fewer features than samples (33 features; 40 samples), which helps reduce the risk of over-fitting.

These resulting 33 PCA features from 40 samples were combined with the following 4 observations from the spectral event analysis of electrode C3: mean event power, mean event F-span, mean event duration, and mean events per epoch, in order to form the final feature set for training and testing our binary classification SVM. We validated the classifier using k-folds cross validation using *k* = 10. Classification accuracy was calculated within the k-folds cross validation by counting the number of out-of-sample predicted labels that matched the true label of the sample, and dividing this total by the number of samples (40, in this case). Receiver Operating Characteristic (ROC) Area Under Curve (AUC) was calculated using the MatLab function *perfcurve*. The SVM hyperparameters box constraint and kernel scale were selected using a grid search. A Gaussian kernel was used. Additionally, 500 iterations of a random permutation procedure were performed, whereby the sample labels were randomly permuted, and then the training and testing process, including the grid search and cross-validation, were repeated using the randomized labels. This process yields a distribution of accuracy and AUC scores, representing the classification performance that could be expected by chance, against which we can evaluate the statistical significance of the classification performance prior to permutation (Ojala and Garriga, 2009; Combrisson and Jerbi, 2015).

Next, we performed these same preprocessing steps (hot deck imputation, PCA) on the 17 pre-SCS samples, using the same PCA coefficients from the radiculopathy/healthy dataset. Note that spectral event features were not included in this dataset, as spectral event analysis was not performed for the SCS group. We then trained and tested a 3-way classification SVM using data from the healthy, radiculopathy and pre-SCS groups. As above, a Gaussian kernel, grid search, and 10-fold cross-validation were used, and accuracy was calculated in the same manner. ROC-AUC was calculated with respect to each of the three groups, yielding 3 AUCs, one with respect to each class. 500 iterations of random shuffling were performed.

## 3. Results

### 3.1. Statistical analysis

**Fig. 1.**
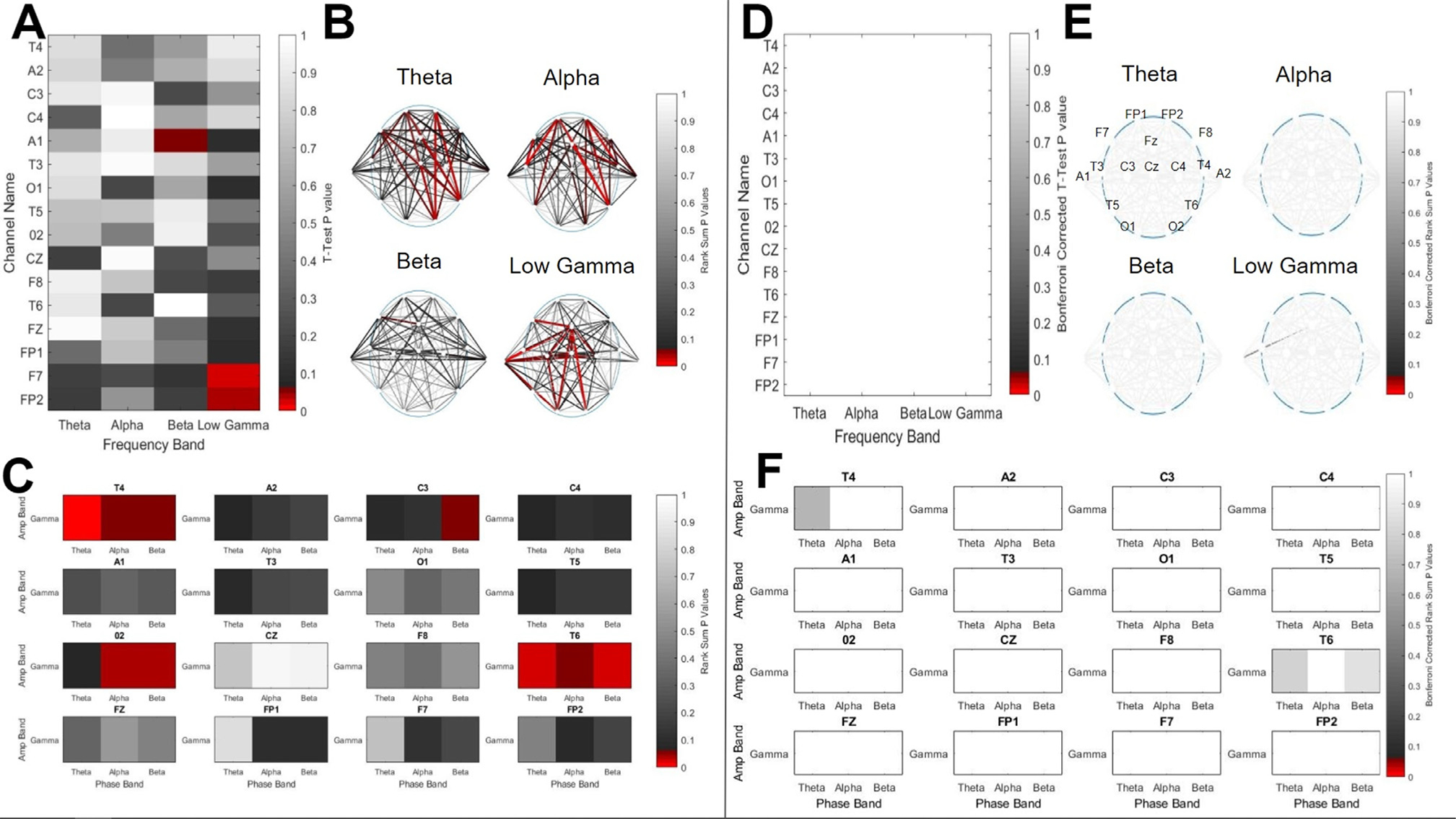
A comparison of EEG bandwise Power Spectral Density (PSD), interchannel coherence, and Phase Amplitude Coupling (PAC) collected from 20 subjects with lumbar radiculopathy, and 20 age and gender matched healthy controls. A) The results of T-Tests comparing the PSDs from the two groups at 16 channels (y-axis) and 4 frequency bands (x-axis). Color represents the significance level of each test, with red cells indicating that the *P* value for that channel and band was < 0.05, and grayscale indicating the opposite, per the color bar. B) The results of rank-sum tests comparing the coherence from the two groups at all unique channel pairs and 4 frequency bands. The color of each line represents the significance level of the test for the two channels connected by the line, with red lines indicating that the *P* value for that channel pair and band was < 0.05, and grayscale indicating the opposite, per the color bar. C) The results of rank-sum tests comparing the PAC from the two groups at 16 channels, as indicated by the subplot titles, between three low frequency bands (Theta, Alpha, and Beta; x-axes) and one high frequency band (Gamma; y-axes). As before, color represents the significance level of each test. D), E), F) These figures represent the same data and results as A), B), and C), respectively, after applying Bonferroni’s correction for multiple comparisons. Additionally, the EEG montage with channel names and locations is shown in subplot E, upper left panel.

Unpaired T-tests comparing bandwise power at each of the 16 channels and 4 bands between the radiculopathy and healthy groups showed significant ( *P* < 0.05) differences in power with the beta band at channel A1, and within low gamma at F7 and FP2 (Fig. 1A). However, each of these differences was found to fail Bonferroni’s correction for multiple comparisons (Fig. 1D). A similar trend was observed both for coherence (Fig. 1B, E) and PAC (Fig. 1C, F), in which significant differences were observed at the *P* < 0.05 level; however, all differences failed Bonferroni’s correction for multiple comparisons. Additionally, no significant differences were found using the Benjamini-Hochberg procedure with a false discovery rate of 0.05. Results from all features for which there were significant differences before correction are summarized in Table 2.

The results of the TOST equivalence tests showed that 48.4% of all PSD features, 67.9% of all coherence features, and 54.2% of all PAC features were found to have means that were significantly equivalent between the radiculopathy and healthy groups to within one standard deviation (Supplementary Figure 1). This shows that for most features, the largest possible effect of group that went unobserved is one standard deviation of the control group

### 3.2. Spectral event analysis

**Fig. 2.**
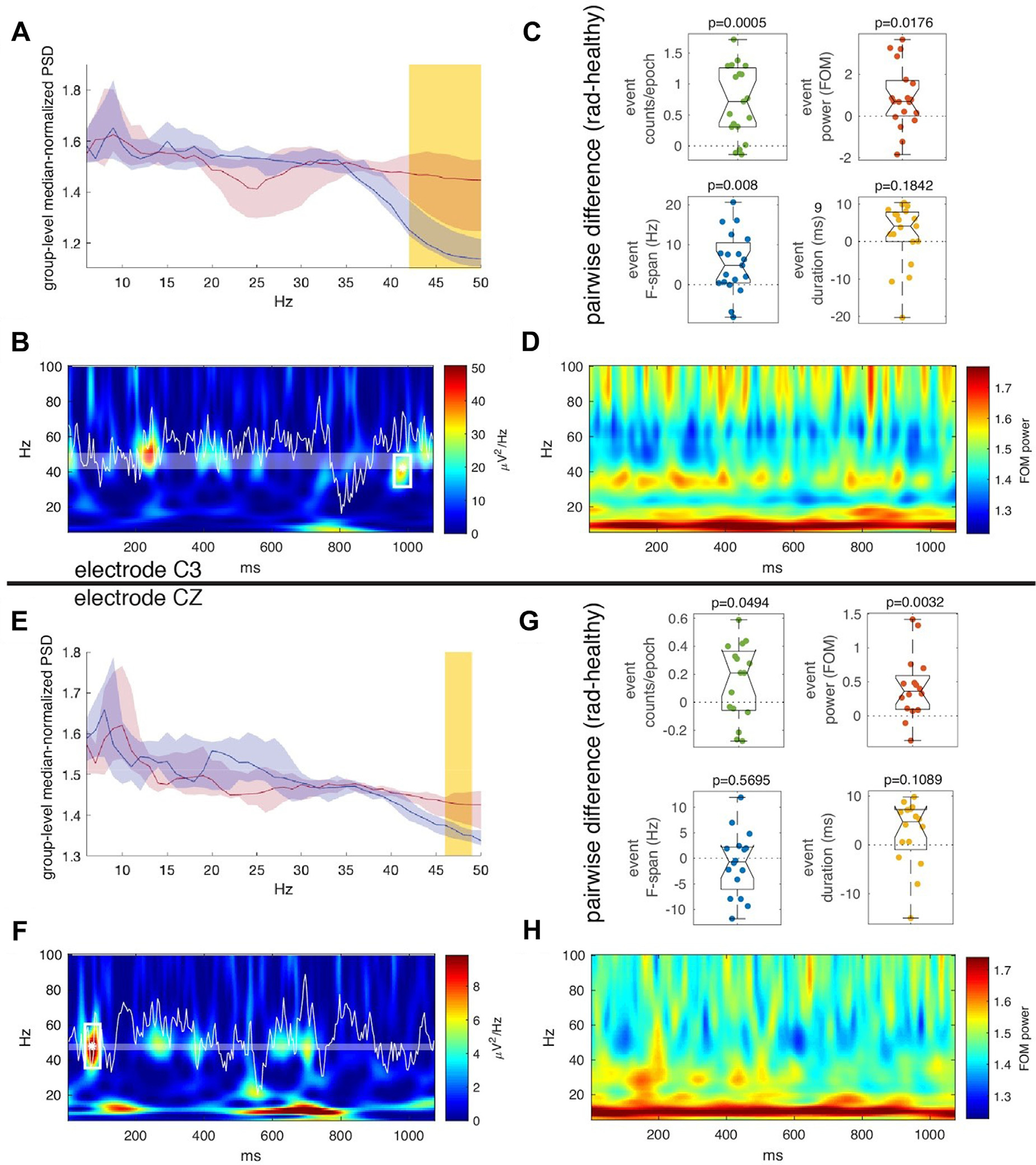
Spectral event analysis in subjects with lumbar radiculopathy, and age and gender matched healthy controls. A-D show analysis of the C3 electrode channel whereas E-F show analysis of the CZ electrode channel. A) Median-normalized power spectral density for radiculopathy subjects (red) was compared to that of healthy subjects (blue) in order to select a unique frequency BOI for each radiculopathy/healthy subject pair. This panel shows results corresponding to a representative example for a pair. Lines show the median +/− bootstrapped 95% confidence interval across the complement set of subjects and the yellow-highlighted frequency band denotes the region of statistically significant difference between the radiculopathy and healthy sets *via* Wilcoxon rank-sum test (p < 0.05, Bonferroni corrected) B) Representative example demonstrating event detection in a single epoch. Shaded area indicates the band of interest, and the asterisk and white rectangle indicate the location of the event found in this epoch within the band of interest. The white waveform is the EEG signal and the heatmap shows the wavelet transform of the signal. C) Comparison of the pairwise difference between radiculopathy and healthy spectral event features to zero (Wilcoxon signed-rank test). The features are as follows: event counts per epoch (green), event power maximum (red), event frequency span (blue), and event duration (yellow). D) Average spontaneous time-frequency response of the radiculopathy group. Note that large amounts of non-time-locked, high amplitude activity occur near the BOI. E-H are similar to A-D except that they represent results from the CZ electrode channel.

BOIs were found for subjects at channel C3 with a lower frequency bound of 40 (40–42) Hz (median and range) and a constant upper frequency bound of 50 Hz in 19 age-matched radiculopathy/healthy subject pairs. BOIs were found for subjects at channel CZ with a lower frequency bound of 46 (43–47) Hz and an upper frequency bound of 48 (44–50) Hz in 16 age-matched radiculopathy/healthy subject pairs. Given that spectral events define sparse topographic regions in the time-frequency domain of high factors-of-the-median (FOM) power, our search for a frequency BOI demonstrated that only one primary frequency band in the 40–50 Hz range differs significantly in high-power spectral fluctuation between the radiculopathy and healthy subjects (Fig. 2A, E). Such activity emerged only from the C3 and CZ electrodes over somatosensory cortex. Furthermore, separation in the 40–50 Hz band between sPSD (spontaneous Power Spectral Density) of the two groups corresponded to a statistically significant difference in spectral event features (Fig. 2B–D, 2F–H). For C3, the pairwise difference between spectral event counts/epoch, power, and F-span in radiculopathy versus healthy subjects deviated significantly from zero, while event duration did not (Fig. 2C). For CZ, the pairwise difference between spectral event counts/epoch and power in radiculopathy versus healthy subjects deviated significantly from zero, while event F-span and duration did not (Fig. 2G). By inspection, it is clear that the radiculopathy subjects demonstrated increased mean spectral event features (i.e., more events, higher power events, and for channel C3, events with a broader spectrum) than their healthy age-matched controls.

### 3.3. Machine learning

**Fig. 3.**
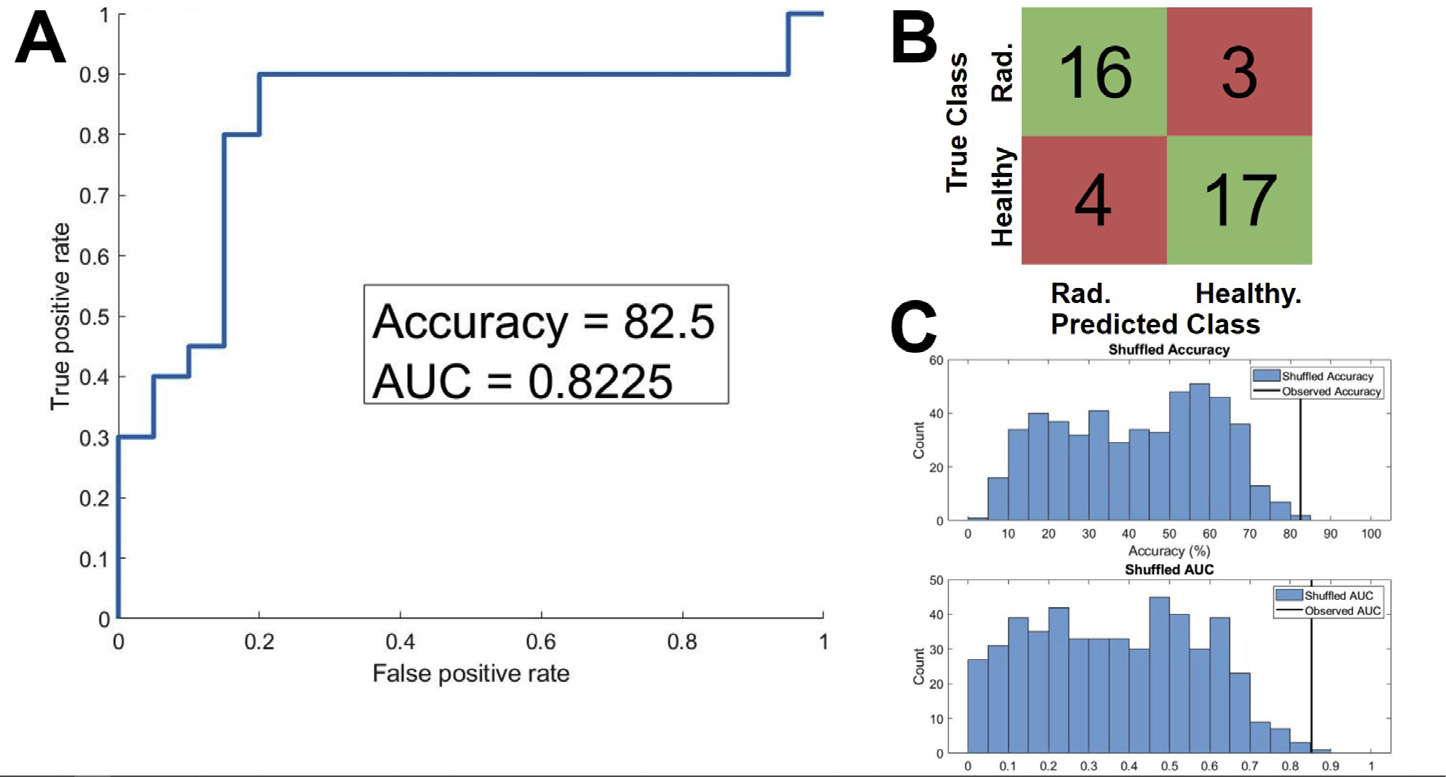
Evaluation of a binary classification support vector machine (SVM) trained to classify between EEGs from subjects with lumbar radiculopathy ( *N* = 20) and aged and gender matched healthy controls ( *N* = 20). A) The Receiver Operating Characteristic (ROC) of the SVM, cross validated using 10-fold cross-validation. The Area Under Curve (AUC) was 0.8225, and the classification accuracy was 82.5%. B) A confusion matrix describing the cross-validation predictions of the trained SVM. 33 out of 40 samples were classified correctly (green squares) and 7 out of 40 were classified incorrectly (red squares). C) The results of 500 iterations of a random shuffling procedure, whereby the labels of the EEGs were randomly reassigned, and the SVM was retrained and retested using the randomized labels. The upper plot shows the histogram of classification accuracy amongst the 500 shuffled iteration in blue, and the true accuracy observed in our classifier in orange (82.5%, as stated above). The observed accuracy was greater than the accuracy all of 500 shuffled iterations, indicating the odds of observing an accuracy of 82.5% given the null hypothesis that the classifier predictions are no better than chance is *P* < 0.002. The bottom shows the same results, except for ROC-AUC. The observed AUC was greater than that of 499 out of 500 shuffled iterations, indicating that *P* = 0.002.

**Fig. 4.**
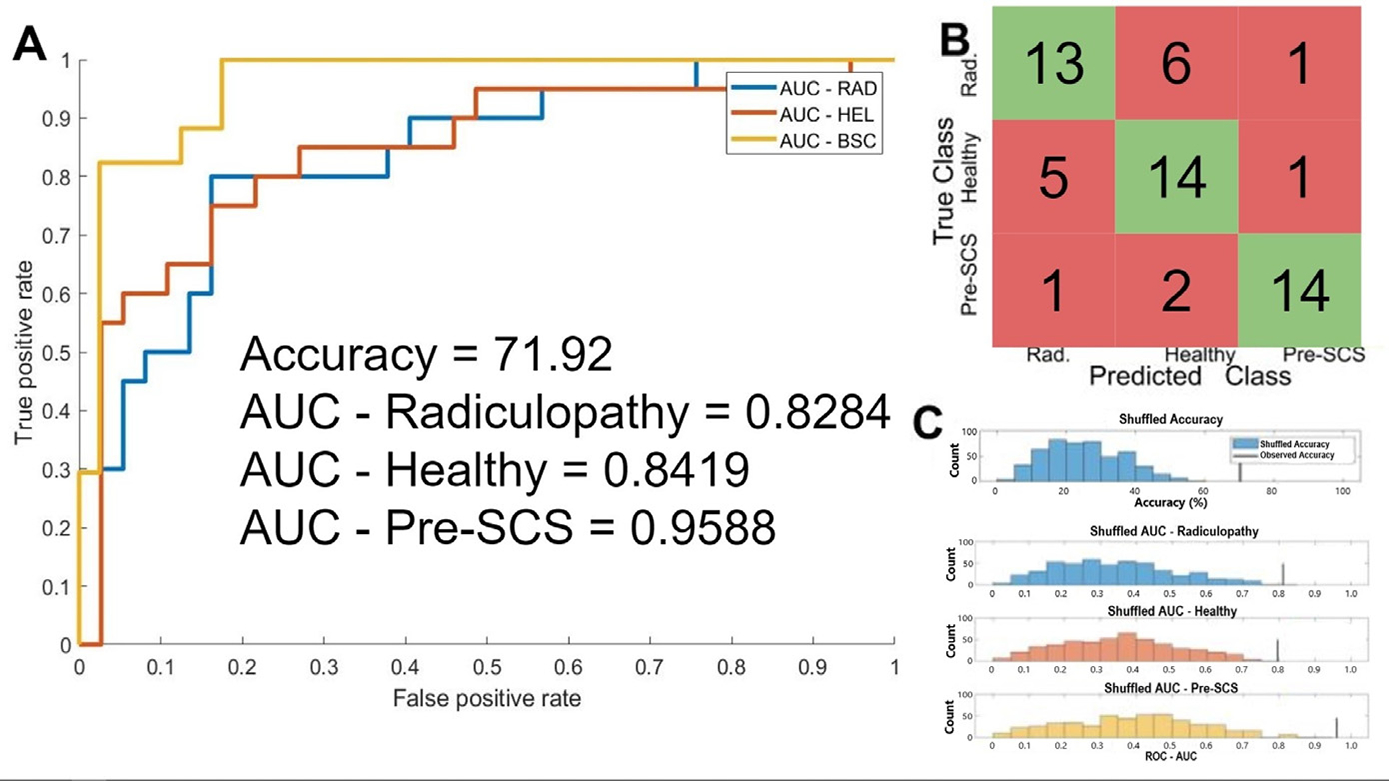
Evaluation of a 3-way classification support vector machine (SVM) trained to classify between EEGs from subjects with lumbar radiculopathy ( *N* = 20), aged and gender matched healthy controls (pre-SCS, *N* = 20), and subjects with chronic back pain who are candidates for spinal cord stimulation ( *N* = 17). A) The Receiver Operating Characteristic (ROC) of the SVM with respect to each of the 3 classes, cross validated using leave-one-out cross-validation. The Area Under Curve (AUC) with respect to the radiculopathy group was 0.828, the AUC with respect to the healthy group was 0.842, the AUC with respect to the chronic pain group was 0.959, and the classification accuracy was 71.9%. B) A confusion matrix describing the cross-validation predictions of the trained SVM. 41 out of 57 samples were classified correctly (green squares) and 16 out of 57 were classified incorrectly (red squares). C) The results of 500 iterations of a random shuffling procedure, whereby the labels of the EEGs were randomly reassigned, and the SVM was retrained and retested using the randomized labels. The upper plot shows the histogram of classification accuracy amongst the 500 shuffled iteration in blue, and the true accuracy observed in our classifier in black (75.4%, as stated above). The observed accuracy was greater than that of all 500 shuffled iterations, indicating the odds of observing an accuracy of 75.4% given the null hypothesis that the classifier predictions are no better than chance is *P* < 0.002. The bottom shows the same results, except for AUC with respect to each class. For the healthy and Pre-SCS class, the observed AUC was greater than that of all of 500 shuffled iterations, indicating that *P* < 0.002. For the radiculopathy class, the observed AUC was greater than that of 499 out of 500 shuffled iterations, indicating that *P* = 0.002.

The binary SVM trained to classify between radiculopathy and healthy subjects was shown to have a cross-validation accuracy of 82.5%, and an ROC-AUC of 0.8225. Random permutation testing found that this accuracy was higher than that of 500 out of 500 iterations, indicating a *P* of < 0.002, and that this ROC-AUC was higher than that of 499 out of 500 iterations, indicating a *P* of 0.002 (Fig. 3). The 3-way classifier had a cross-validation accuracy of 71.9%, an AUC-radiculopathy of 0.828, an AUC–Healthy of 0.842, and an AUC-pre-SCS of 0.962. Random permutation testing of the 3-way classifier showed that the accuracy, AUC–Healthy and AUC-Pre-SCS were greater than the corresponding values of all 500 shuffled iterations, indicating *P* < 0.002 in each case, and the AUC-Radiculopathy was greater than the corresponding value from 499 out of 500 iterations, indicating *P* = 0.002 (Fig. 4). We therefore conclude that the performance of both classifiers is significantly higher than would be expected by chance alone.

## 4. Discussion

Our results demonstrate that after carefully accounting for variables including automated detection of EEG artifacts, age and gender bias, and correction methods for multiple comparisons, no statistically significant difference was found in the mean resting state EEG power between healthy subjects and pain patients within common frequency bands. Moreover, there was no statistically significant difference in paired channel coherence or PAC. This is further supported by the many significant results of the equivalence tests, which supports a hypothesis that there is not a difference between the two groups, or that the difference is small. Generally, there is a lack of consensus regarding the frequency band(s) altered during pain, the direction of the modulatory effects, and the cortical areas involved (Pinheiro et al., 2016). Our results stand in contrast to previous studies suggesting enhanced resting state EEG power in pain patients within the theta (Schulman et al., 2005; Stern et al., 2006), alpha (van den Broeke et al., 2013), and beta (Sarnthein et al., 2006) bands. They also contradict previous work that showed modulation of interchannel coherence in individuals with migrain (Mendonça-de-Souza et al., 2012). Changes in the PAC feature of the EEG has not been previously investigated in pain patients. Although noxious laser stimuli have been shown to evoke theta-gamma coupling in rats (Wang et al., 2011), it is not clear how these findings relate to EEG in humans with chronic pain.

Another important factor to consider is the pain state as a variable. The effects of experimentally-induced tonic versus phasic pain on EEG (Hadjileontiadis, 2015; Levitt et al., 2017; Misra et al., 2017; Vijayakumar et al., 2017), as well as resting state versus evoked response potentials (ERP) (Schulz et al., 2012; Huang et al., 2013; Bai et al., 2016) further clouds the relationship between clinical pain and neocortical oscillations, given that phasic, tonic and chronic pain may have distinct physiological patterns (Apkarian et al., 2009; Guo et al., 2016). Other variables that should be controlled for in an EEG study are age and gender. Both age (Hughes and Cayaffa, 1977; Nguyen et al., 2013; Vlahou et al., 2014; Zoubi et al., 2018) and gender (Hu, 2018; van Putten et al., 2018) have been found to impact EEG. Since chronic pain may be more prevalent in one gender, or in specific age cohorts (Elzahaf et al., 2016; Malon et al., 2018), there is a risk of creating a sampling bias if these factors are not controlled for. Ideal EEG studies for chronic pain should be conducted with a dataset in which chronic pain subjects are paired with age and gender matched healthy controls, or at least a dataset with demographics that are not significantly different between the two groups.

Moreover, some previous studies used a high dimensional data set, but did not correct for multiple comparisons, which presumably contributed to Type I errors (Bjørk et al., 2009; Jensen et al., 2013). This could, in turn, contribute to the diversity of results in the pain EEG literature (Pinheiro et al., 2016). Type 1 errors are ‘false positive’ errors, when a statistical test shows a significant result when in fact the null hypothesis is correct. The risk of encountering this type of error increases as the number of statistical tests increases. This is typically controlled for by using a correction for multiple comparisons, such as the Bonferroni test; however, these corrections come with their own risks, as they increase the risk of type 2 errors, making it harder to detect genuine differences. This can be a challenging problem to solve, especially when the number of tests is great, as is often the case in EEG studies. A conservative approach would be to apply the correction, and accept the associated risk of false negatives (Curran-Everett, 2000), as in this study. These concerns could be partly addressed by increasing statistical power, for example by increasing sample size. While the sample size of this study is comparable to that of other studies of EEG and pain (Pinheiro et al., 2016), future research could benefit from an increased sample size, perhaps by performing multi-site recruitment.

Interestingly, we report that transient low gamma events are altered in pain patients, specifically in a band from 42 to 43 Hz. In particular, we found that subjects with radiculopathy manifest more of these events per 1 second epoch compared to healthy subjects. Since the aggregate of radiculopathy features appeared to have greater power and greater F-span (but did not significantly separate the radiculopathy from healthy subjects with average event power or average event F-span on a subject-by-subject basis), the statistical significance of these two features observed in Fig. 2D, F was mostly likely introduced by only a few subjects (i.e., these features do not represent population-level differences between the two groups). Furthermore, we did not find any difference in the duration of events between the radiculopathy and healthy groups. This highlights the importance of investigating transient changes in power at the sub-second scale, whereas others typically study power in relatively long, averaged epochs of variable lengths (Sarnthein et al., 2006; Stern et al., 2006; Jensen et al., 2013; Misra et al., 2017). It has been demonstrated that beta (15–29 Hz) transients in S1 occurring in temporal proximity to a sensory stimulus are more likely to impair perception based on magnetoencephalography (MEG) data in humans and local field potential recordings in mice performing a detection task, such that increased rate of beta events predicts failure to effectively transmit information through specific neocortical representations (Shin et al., 2017). There is also evidence that gamma oscillations in S1 are linked to nociceptive pathways (Tan et al., 2019). The functional significance of altered gamma transients in our study remains unknown. One option to elucidate this phenomenon would be to explore the behavioral consequences of optogenetic, time-locked modulation of gamma bursts in S1, as has been previously demonstrated for longer-duration theta oscillations (LeBlanc et al., 2017).

Lastly, we demonstrate that machine learning using principal components is a valid approach for developing ‘classifier’ algorithms that accurately distinguish between healthy ‘no-pain’ subjects and those with ‘high-pain.’ Moreover, we show that a 3-way classifier accurately distinguishes between different ‘high-pain’ states in patients. This demonstrates that, despite the lack of significant differences between the healthy and radiculopathy groups based on conventional analysis of EEG data, principal components derived from this dataset contain physiologically relevant information pertaining to sensory states, and that this information may be used to help distinguish between patient populations when other clinical signs are inconclusive or ambiguous. It should be noted, however, that a different amplifier from the same manufacturer with a higher sample rate was used to collect the Pre-SCS EEGs. This was corrected for using filtering and downsampling. Communication with the manufacturer indicated that the two devices were essentially identical, except for their sampling rate. Additionally, the EEG data for the statistical analysis, spectral event analysis, and the two-way classifier were all collected using only one amplifier.

Of note, pain intensity scores in both pain groups were similar, but the clinical diagnosis of subjects in either group was distinct. Patients in the treatment-resistant chronic back pain group (the Pre-SCS group), but not those in the radiculopathy pain group, were diagnosed by a board-certified pain physician to be clinically appropriate for pain management by SCS. This is a critical point because the go/no-go decision to implant SCS leads is based mostly on subjective criteria, in addition to being time consuming, costly and emotionally onerous for the patient (Campbell et al., 2013)

Determining the contribution or importance of each feature to the performance of the classifiers is made intractable by two processes: 1) Conversion from the original feature space into principal component space. This means that each individual original feature contributes, to some extent, to each PC feature; 2) nonlinearity of a gaussian SVM. This means that individual features are not assigned a simple weight, as they would be for a linear SVM. Furthermore, the decision regions created by a gaussian SVM may be discontinuous, tortuous, or both. Thus, interpretation of the impact of individual features on classification is difficult to discern without further investigation.

High dimensional data representing hundreds of power, coherence, and PAC features generated by multi-channel EEG systems require advanced statistical tools beyond conventional first order statistics. Machine learning methods can help detect patterns that may otherwise go undetected, and improve performance in predictive tasks (Bzdok et al., 2018). These algorithms have already been applied in similar contexts, such as detecting traumatic brain injury (Prichep et al., 2014), estimating depth of anesthesia (Saadeh et al., 2019), studying Alzheimer’s disease (Simpraga et al., 2017), and studying seizure activity (Elahian et al., 2017). There is also evidence for using EEG data and an SVM method to predict the diagnosis of complex and variable neurodevelopmental conditions, such as autism spectrum disorder (Bosl, 2018; Bosl et al., 2018). Recently, machine learning methods have produced valid and reliable algorithms for predicting pain intensity using high dimensional EEG features. For example, Misra et al. developed a classifier for high/low experimentally-induced pain using theta, beta, and gamma mean power (Misra et al., 2017), and Vuckovic et al. did the same for a subacute pain model (Vuckovic et al., 2018), whereas Vanneste et al. distinguished between pain and other neurological conditions using standardized low-resolution brain electromagnetic tomography (Vanneste et al., 2018). Shen et al., 2019 also used an SVM model to analyze BOLD fMRI data and distinguish between patients with chronic low back pain and controls (Shen et al., 2019). At the time of submission of our manuscript, Dinh et al. published a study similar to ours using resting state EEG features to classify chronic pain patients and healthy controls with 57% accuracy; albeit with broader inclusion criteria. Additionally, they also did not identify significant differences between healthy and pain groups using first-order EEG features, which concurs with our results. (Dinh et al., 2019). Additional layers of complexity or nonlinearity could be introduced in various ways, for example by using a nonlinear dimensionality reduction technique, such as minimum curvilinear embedding (Cannistraci et al., 2013). These efforts are important steps, both broadly toward a better understanding of the physiology of pain and sensory perception, and toward the specific goal of developing objective biosignatures for pain, according to guidelines that have been recently reviewed by Davis et al., press).

Phenotypic categorization of pain according to distinct disease classifications, based on a relatively inexpensive, rapid and objective EEG ‘screen,’ is highly relevant and urgently needed in clinical settings. Further refinement, testing and validation of our SVM algorithms in a larger sample size would be required to reach this goal. EEG analysis is high dimensional, and the applicable types of analyses are myriad, which can make determining the best way forward challenging, but nonetheless feasible and clinically valuable.

## Supplementary Material

Supplementary Material

## Figures and Tables

**Table 1:** Demographic information for age matched radiculopathy subjects and healthy controls, and for spinal cord stimulation subjects.

Pair No.	Radiculopathy Subjects				Healthy Controls	
	Age (years)	Gender	Visual Analog Score (VAS)	Duration of pain (years)	Age (years)	Gender

**1**	62	F	7	3	63	F
**2**	60	F	10	6	60	F
**3**	72	M	7	1.1	70	M
**4**	40	F	5	0.5	39	F
**5**	32	F	6	7	30	F
**6**	53	M	8	>10	56	M
**7**	52	F	9	>10	55	F
**8**	60	M	6	3	62	M
**9**	68	F	7	>10	63	F
**10**	72	F	7	8	70	F
**11**	44	M	8	>10	45	M
**12**	80	F	8	1	82	F
**13**	70	F	6	2	69	F
**14**	63	M	6	>10	63	M
**15**	35	M	9	0.5	37	M
**16**	51	F	6	5	48	F
**17**	46	M	6	4	46	M
**18**	47	F	8	5	47	F
**19**	50	M	5	3	51	M
**20**	28	M	8	6	27	M
**MEAN**	54.25		7.1		54.15	
Subject No.	Subject Candidates for Spinal Cord Stimulation					
**1**	79	M	3			
**2**	41	F	7			
**3**	65	F	10			
**4**	41	F	7			
**5**	72	F	7			
**6**	72	M	8			
**7**	33	F	5			
**8**	55	M	9			
**9**	61	M	7			
**10**	36	F	7			
**11**	57	F	8			
**13**	51	M	7			
**14**	64	F	8			
**16**	43	F	9			
**17**	60	M	8			
**18**	58	F	8			
**19**	79	M	9			
**MEAN**	56.88		7.47			

**Table 2:** EEG Features for which there was a significant difference.

Channel	Frequency Band	Rad. Average	Healthy Average	P-value

Power Spectral Density				
A1	Beta	0.0145	0.0118	0.0494
F7	Low Gamma	0.0127	0.0067	0.0235
FP2	Low Gamma	0.0137	0.0088	0.0401
Coherence				
A2-CZ	Beta	0.247	0.209	0.0443
FZ-F7	Beta	0.244	0.290	0.0332
C3-C4	Gamma	0.3265	0.4091	0.0290
C3-A1	Gamma	0.2758	0.3534	0.0210
C3–O1	Gamma	0.3253	0.4114	0.0180
C3–O2	Gamma	0.2662	0.3147	0.0420
C3-FZ	Gamma	0.2046	0.2451	0.0110
C4-C3	Gamma	0.3265	0.4091	0.0290
C4-A1	Gamma	0.2629	0.3299	0.0210
C4-FZ	Gamma	0.2125	0.2472	0.0180
A1-O1	Gamma	0.3069	0.3856	0.0140
A1-CZ	Gamma	0.2415	0.2851	0.0270
A1-FZ	Gamma	0.1972	0.2359	0.0010
O1-A1	Gamma	0.3069	0.3856	0.0140
O1-FZ	Gamma	0.1985	0.2323	0.0140
O2-FZ	Gamma	0.1913	0.2236	0.0060
CZ-FZ	Gamma	0.1905	0.2197	0.0170
F8-FP1	Gamma	0.4033	0.3579	0.0440
FZ-F7	Gamma	0.2111	0.2354	0.0120
A2-FZ	Theta	0.2208	0.2429	0.0540
A2-F7	Theta	0.2777	0.3270	0.0390
A2-FP2	Theta	0.2526	0.3106	0.0440
A1-CZ	Theta	0.2771	0.3287	0.0500
T3-FP2	Theta	0.2765	0.3338	0.0310
O2-F8	Theta	0.2375	0.2749	0.0320
O2-FP1	Theta	0.2085	0.2555	0.0100
O2-F7	Theta	0.2236	0.2625	0.0280
O2-FP2	Theta	0.2031	0.2427	0.0160
F8-T6	Theta	0.2927	0.3534	0.0460
F8-F7	Theta	0.3289	0.3651	0.0390
T6-FZ	Theta	0.2055	0.2248	0.0340
T6-FP1	Theta	0.2297	0.3030	0.0100
T6-F7	Theta	0.2350	0.2881	0.0210
T6-FP2	Theta	0.2143	0.2741	0.0140
T4-F8	Alpha	0.4071	0.4930	0.0340
T4-FZ	Alpha	0.2024	0.2372	0.0120
T4-FP1	Alpha	0.2874	0.3606	0.0070
A2-F8	Alpha	0.3767	0.4474	0.0400
A2-FZ	Alpha	0.2007	0.2318	0.0360
A2-FP1	Alpha	0.2812	0.3524	0.0290
A2-FP2	Alpha	0.2435	0.3063	0.0490
A1-FP1	Alpha	0.2243	0.2586	0.0500
T3-F7	Alpha	0.3858	0.4882	0.0050
T3-FP2	Alpha	0.2627	0.3447	0.0050
TS-FP1	Alpha	0.1954	0.2470	0.0350
T5-FP2	Alpha	0.2100	0.2783	0.0240
O2-FP1	Alpha	0.2012	0.2347	0.0320
O2-FP2	Alpha	0.1970	0.2311	0.0370
F8-T6	Alpha	0.2887	0.3506	0.0400
T6-FZ	Alpha	0.1880	0.2179	0.0120
T6-FP1	Alpha	0.2206	0.2911	0.0140
T6-F7	Alpha	0.2173	0.2647	0.0530
T6-FP2	Alpha	0.2019	0.2669	0.0050
Phase-Amplitude Coupling				
T4	Gamma vs. Theta	2.44e-04	1.46e-04	0.0141
	Gamma vs. Alpha	l.22e-04	9.22e-05	0.0541
	Gamma vs. Beta	9.20e-05	6.85e-05	0.0541
C3	Gamma vs. Beta	8.34e-05	6.20e-05	0.0565
O2	Gamma vs. Theta	3.91e-04	2.44e-04	0.0634
	Gamma vs. Alpha	1.98e-04	1.19e-04	0.0317
	Gamma vs. Beta	1.38e-04	8.66e-05	0.0452
T6	Gamma vs. Theta	3.33e-04	2.20e-04	0.0167
	Gamma vs. Alpha	1.58e-04	1.21e-04	0.0493
	Gamma vs. Beta	1.16e-04	7.65e-05	0.0182

Each row of the table contains the feature name, group averages, and P value, for each statistical test which had a *P* < 0.05 before applying Bonfcrroni’s correction for multiple comparisons. No features had *P* < 0.05 after applying Bonfcrroni’s correction. T-tests were used for power spectral density features, and rank-sum tests were used for coherence and phase-amplitude coupling features.
