# Supplementary material for: Pain phenotypes classified by machine learning using electroencephalography features
Source: Neuroimage. Author manuscript; Available in PMC 2022 May 9. (PMC9084327; doi:10.1016/j.neuroimage.2020.117256)
Supplement: Supplementary Material [file NIHMS1797911-supplement-Supplementary_Material.docx]

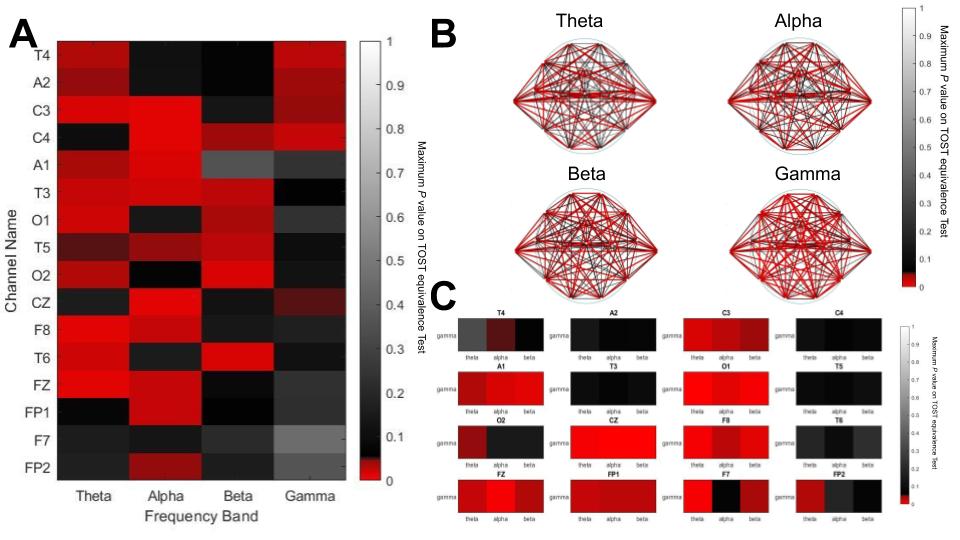


Supplementary Figure 1. A comparison of bandwise Power Spectral Density (PSD), interchannel coherence, and Phase Amplitude Coupling (PAC) collected from EEGs from 20 subjects with lumbar radiculopathy, and 20 age and gender matched healthy controls. A) The results of TOST equivalence tests, with an equivalence interval of +/- one standard deviation of the healthy distribution for each feature, comparing the PSDs from the two groups at 16 channels (y-axis) and 4 frequency bands (x-axis). Color represents the significance level of each test, with red cells indicating that the *P* value for that channel and band was < 0.05, and grayscale indicating the opposite, per the color bar.  B) The results of TOST equivalence tests, with the same equivalence interval as in A), comparing the coherence from the two groups at all unique channel pairs and 4 frequency bands. The color of each line represents the significance level of the test for the two channels connected by the line, with red lines indicating that the *P* value for that channel pair and band was < 0.05, and grayscale indicating the opposite, per the color bar. C)  The results of TOST equivalence tests, with the same equivalence interval as in A), comparing the PAC from the two groups at 16 channels, as indicated by the subplot titles, between three low frequency bands (Theta, Alpha, and Beta; x-axes) and one high frequency band (Gamma; y-axes). As before, color represents the significance level of each test.

Supplementary Table 1. Percent of all epochs accepted by automated artifact detection from each channel for each of the three experimental groups

| Channel | % epochs accepted | | |
| --- | --- | --- | --- |
|  | Healthy | Radiculopathy | SCS |
| 'T4' | 78 | 77 | 69 |
| 'A2' | 68 | 67 | 66 |
| 'C3' | 81 | 80 | 66 |
| 'C4' | 77 | 76 | 68 |
| 'A1' | 65 | 64 | 64 |
| 'T3' | 76 | 76 | 67 |
| 'O1' | 76 | 76 | 67 |
| 'T5' | 72 | 71 | 65 |
| '02' | 76 | 75 | 63 |
| 'CZ' | 76 | 75 | 69 |
| 'F8' | 51 | 50 | 60 |
| 'T6' | 75 | 75 | 66 |
| 'FZ' | 46 | 45 | 52 |
| 'FP1' | 37 | 36 | 51 |
| 'F7' | 51 | 51 | 53 |
| 'FP2' | 36 | 35 | 48 |
